# Universal masking to control healthcare-associated transmission of severe acute respiratory coronavirus virus 2 (SARS-CoV-2)

**DOI:** 10.1017/ice.2021.127

**Published:** 2021-03-29

**Authors:** Eliza R. Thompson, Faith S. Williams, Pat A. Giacin, Shay Drummond, Eric Brown, Meredith Nalick, Qian Wang, Jay R. McDonald, Abigail L. Carlson

**Affiliations:** 1 Washington University School of Medicine, St Louis, Missouri; 2 Department of Infection Prevention, Veterans’ Affairs (VA) St Louis Health Care System, St Louis, Missouri; 3 Infectious Diseases Section, VA St. Louis Health Care System, St Louis, Missouri; 4 Division of Infectious Diseases, Allergy and Immunology, St Louis University, St Louis, Missouri; 5 Division of Infectious Diseases, Washington University School of Medicine, St. Louis, Missouri

## Abstract

**Objectives:**

To assess extent of a healthcare-associated outbreak of severe acute respiratory coronavirus virus 2 (SARS-CoV-2) and to evaluate the effectiveness of infection control measures, including universal masking.

**Design:**

Outbreak investigation including 4 large-scale point-prevalence surveys.

**Setting:**

Integrated VA healthcare system with 2 facilities and 330 beds.

**Participants:**

Index patient and 250 exposed patients and staff.

**Methods:**

We identified exposed patients and staff and classified them as probable and confirmed cases based on symptoms and testing. We performed a field investigation and an assessment of patient and staff interactions to develop probable transmission routes. Infection prevention interventions included droplet and contact precautions, employee quarantine, and universal masking with medical and cloth face masks. We conducted 4 point-prevalence surveys of patient and staff subsets using real-time reverse-transcriptase polymerase chain reaction for SARS-CoV-2.

**Results:**

Among 250 potentially exposed patients and staff, 14 confirmed cases of coronavirus disease 2019 (COVID-19) were identified. Patient roommates and staff with prolonged patient contact were most likely to be infected. The last potential date of transmission from staff to patient was day 22, the day universal masking was implemented. Subsequent point-prevalence surveys in 126 patients and 234 staff identified 0 patient cases and 5 staff cases of COVID-19, without evidence of healthcare-associated transmission.

**Conclusions:**

Universal masking with medical face masks was effective in preventing further spread of SARS-CoV-2 in our facility in conjunction with other traditional infection prevention measures.

Since its emergence in China in December 2019, severe acute respiratory coronavirus virus 2 (SARS-CoV-2) has caused an unprecedented global pandemic.^1–3^ As of December 22, 2020, nearly 18 million cases of coronavirus disease 2019 (COVID-19) had been identified in the United States alone.^4^ Although SARS-CoV-2 is known to spread person to person, exact modes of transmission remain a topic of debate.^5,6^

A growing body of evidence suggests that transmission occurs primarily via respiratory droplets. Although studies showing viable viral RNA on shared surfaces and in smaller aerosols raise concerns for fomite and airborne transmission,^7,8^ their contribution to the overall spread of disease remains unknown. Amid this uncertainty, many healthcare facilities have turned to universal masking protocols, in which most or all individuals entering a facility are required to wear a mask as one means of preventing spread of SARS-CoV-2.^9,10^

Previous small randomized controlled trials have demonstrated reduced transmission of respiratory illness after universal masking implementation within hospital units.^11,12^ More recently, a meta-analysis of data from 39 studies of severe acute respiratory syndrome (SARS), Middle East respiratory syndrome (MERS), and SARS-CoV-2 outbreaks revealed a large reduction in risk of infection among exposed individuals using face masks or N95 respirators.^13^ However, further data on facility-wide protocols are needed to assess the impact of universal masking on transmission within healthcare settings.

Here, we describe infection control policies implemented in response to an outbreak of COVID-19 at a Veterans’ Affairs (VA) healthcare system, including institution-wide universal masking. We subsequently evaluate the effect of universal masking on healthcare-associated SARS-CoV-2 transmission using data from institutionally mandated point-prevalence surveys.

## Identification of the outbreak

Our health system has 2 highly integrated facilities, with regular inpatient transfers and patient and staff travel between them. Facility 1 (112 beds) houses 2 intensive care units, a step-down unit, 3 general medical-surgical inpatient wards, and outpatient clinics. Facility 2 (218 beds) includes outpatient clinics; community living center (CLC) units housing extended care, rehabilitation and hospice patients; spinal cord injury (SCI) units; and mental health units.

The index patient (patient A) was a man with diabetes mellitus type 2 and an active cancer who was undergoing rehabilitation at facility 2 following hospitalization at facility 1. Patient A traveled regularly to facility 1 for appointments at a procedural clinic. On day 1 of the outbreak, a staff member at this clinic returned to work after out-of-state travel with fatigue, cough, fever, and myalgia. This staff member was not wearing a mask or respirator at the time of interaction with patient A. Several clinic staff members subsequently developed similar symptoms.

On outbreak day 7, patient A developed fever and received intravenous antibiotics for a suspected urinary tract infection. A subsequent chest radiograph showed bilateral lower lobe interstitial infiltrates. After continued fever, he was transferred on day 9 to the emergency department at facility 1. Blood cultures and a respiratory viral panel were negative. A nasopharyngeal swab for SARS-CoV-2 real-time reverse-transcriptase polymerase chain reaction (rRT-PCR) testing was sent to the state public health department because in-house testing was unavailable. He was admitted to the unit designated for ruling out COVID-19 on airborne and contact precautions. Because he remained afebrile overnight and without respiratory symptoms, the source of his fever was presumed to be catheter-associated urinary tract infection. He was transferred to a general inpatient ward on day 10 without isolation precautions. On day 13, his SARS-CoV-2 test returned positive, and he was transferred to a designated COVID-19 ward. Because he had been at facility 2 for 22 days prior to symptom onset, his case was classified as healthcare associated.

A second patient admitted on day 3 to the general inpatient ward (patient C) developed cough on day 15. On day 18, he developed fever, prompting SARS-CoV-2 testing, which returned positive that day. This was the second identified case of healthcare-associated COVID-19, and the first at facility 1, which prompted an intensive outbreak investigation.

## Methods

### Case definitions

Exposure was determined by the presence of a known COVID-19–positive individual in a given facility location. Infectivity was calculated using an incubation period of 1–14 days prior to symptom onset (or prior to first positive test if asymptomatic). Patients were considered exposed if they had an appointment at the procedural clinic during days 1–25 or had been admitted to the general inpatient ward during days 10–40. Staff were considered exposed if they worked in the procedural clinic during days 1–25 or in the general inpatient ward during days 10–40. Patients and staff not in those locations but with unmasked contact with a confirmed positive case at either facility were also considered exposed.

Major criteria (subjective or measured fever, cough, shortness of breath) and minor criteria (chills, fatigue, myalgias, headache, anosmia, sore throat, gastrointestinal symptoms, imaging demonstrating bilateral infiltrates) were determined based on Centers for Disease Control and Prevention (CDC) guidance and early descriptive reports of disease from China.^14–16^ Cases were deemed “probable” if they met 2 major criteria or 1 major and 1 or more minor criteria, regardless of testing status. Cases were deemed “confirmed” if they had a positive SARS-CoV-2 result by real-time reverse-transcriptase polymerase chain reaction (rRT-PCR). Due to limited testing supplies, we were unable to test asymptomatic patients and staff outside the point-prevalence surveys or unless necessary for transfer to an outside facility.

### Chart review, contact tracing, and field investigation

Patient medical records were reviewed, and cases were classified according to the definitions above. Clinical data on symptom onset, exposures, testing results, and outcomes were compiled using REDcap (Research Electronic Data Capture) tools hosted at the Veterans’ Health Administration (VHA). REDcap is a secure, web-based software platformed designed to support data capture and management.^17,18^ Staff exposed to confirmed cases were excluded from work for 14 days after last known exposure and were asked to self-quarantine at home while monitoring symptoms. Staff symptom and testing data were obtained from the institution’s employee health department. The most likely routes of transmission were reconstructed, assuming an incubation period of 1–14 days.^19^

Physical spaces involved in the outbreak (ie, procedural clinic, support staff gathering area, and an inpatient room where multiple transmission events occurred) were investigated, and relevant measurements were obtained.

### Interventions

**Fig. 1.**
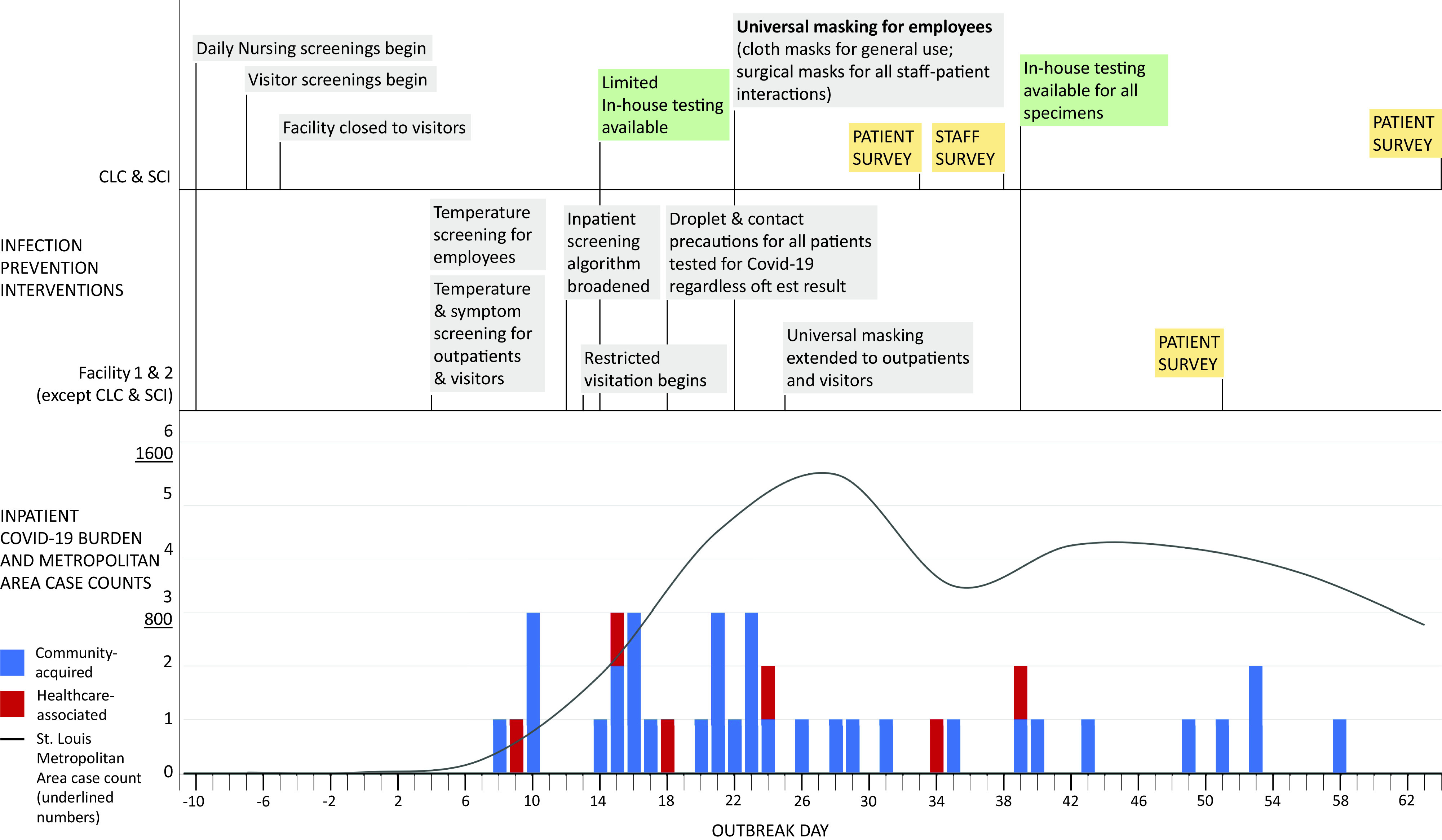
Timeline of infection prevention interventions implemented across both campuses. The x-axis shows outbreak days. The graph shows laboratory confirmed inpatient healthcare-associated (red) and community-associated (blue) cases of COVID-19 by date of test collection, as well as weekly case counts in the St Louis Metropolitan Area. Healthcare-associated cases were defined by symptoms arising >72 hours after admission. The St Louis Metropolitan Area includes St Louis City, St Louis County, and St Charles County in Missouri and Madison County, Monroe County, and St Clair County in Illinois.

Infection prevention policies were implemented in response to increasing community prevalence of COVID-19 and the facility 1 outbreak (Fig. 1). Throughout the outbreak, community case counts increased from ∼50 new cases per day on day zero to a peak of ∼1,600 cases per day on day 26 and remained at ∼1,000 cases per day after day 38. Nursing staff at both facilities began screening inpatients for COVID-19 symptoms 11 days before the outbreak. Visitor screening at the CLC and SCI unit began 7 days prior to the outbreak, with closure to nonessential visitors 5 days prior. Screening at facilities 1 and 2 entrances began on outbreak day 4. Employees were screened for temperature only, while patients and visitors were also asked about respiratory symptoms. Inpatients and new admissions who screened positive and had a SARS-CoV-2 test sent remained on droplet and contact precautions until results were available or an alternative diagnosis was identified. All visitors to both facilities were restricted beginning day 12.

On day 13, the inpatient COVID-19 screening algorithm was broadened to test all patients with fever, new cough, or shortness of breath plus 1 minor symptom (as defined previously), and droplet and contact precautions could be discontinued only with a negative COVID-19 result. Beginning day 18, all tested patients were placed on droplet and contact precautions upon testing, regardless of result. Universal masking among staff was implemented on day 22, and medical masks were required for patient interaction and cloth masks at all other times, including in offices, lobbies, etc. The policy was extended to outpatients and visitors on day 25. Eye protection was permitted but not mandated throughout the study period. All new admissions were tested starting on day 50.

### Surveillance

Infection prevention staff maintained a database of tested patients within our healthcare system as required for public health reporting. The employee health department maintained similar data on employees.

Patient cases were defined as healthcare associated if symptoms began >72 hours after admission. All cases not meeting this definition were considered community acquired. Employee cases were defined as healthcare associated if symptoms began within 1–14 days after unmasked workplace exposure to a known COVID-19–positive case.

We conducted 4 point-prevalence surveys on patients and staff beginning on day 33 (Table 1). Surveys 1, 2, and 4 were performed in response to VHA directives requiring screening for CLC and SCI patients and staff at facility 2; these did not include facility 1. Survey 3 was conducted across all residential and inpatient units at both facilities, except the CLC and the SCI unit, before the adoption of universal testing for new admissions. Testing for surveys 1 and 2 was provided by the VA Palo Alto Public Health Laboratory. Testing for surveys 3 and 4 was performed in house using the BD MAX System rRT-PCR (Becton Dickinson Diagnostic Systems, Sparks, MD) with primers developed by the CDC and manufactured by Integrated DNA Technologies (Coralville, IA).

**Table 1.** Summary of Point-Prevalence Surveys

Survey No.	Day(s)	Population Tested	Units Tested	Exclusions	Positive Results	Negative Results
1^a^	33	Patients (n = 33)	CLC (n = 18) SCI (n = 15)	None	0	33
2^a^	38–41	Staff (n = 234)	CLC (n = 40) SCI (n = 107) Unassigned (n = 87)	Staff not working on campus due to telework or extended leave (n = 40)	5	229
3^b^	50–51	Patients (n = 67)	Facility 1 • General inpatient wards (n = 23) • ICU (n = 7) Facility 2 • Residential rehabilitation program (n = 27) • Mental health inpatient wards (n = 23)	COVID-19 wards, the CLC and the SCI unit, and patients tested in the prior 72 h	0	67
4^b^	65	Patients (13)	CLC (n = 13)	Patients tested in the prior 72 h	0	13

Note. CLC, community living center; ICU, intensive care unit; SCI, spinal cord injury unit; VA, Veterans’ Affairs.

a Tests performed by VA Palo Alto Public Health Laboratory.

b Tests performed by study health system in-house laboratory.

### Study oversight

All interventions were conducted as part of normal institutional operations for outbreak control and infection prevention. Prior to manuscript preparation, the VA St Louis Health Care System Research Office determined that this study met the definition of nonresearch operations activities in accordance with VHA Program Guide 1200.21.

## Results

### Outbreak

**Fig. 2.**
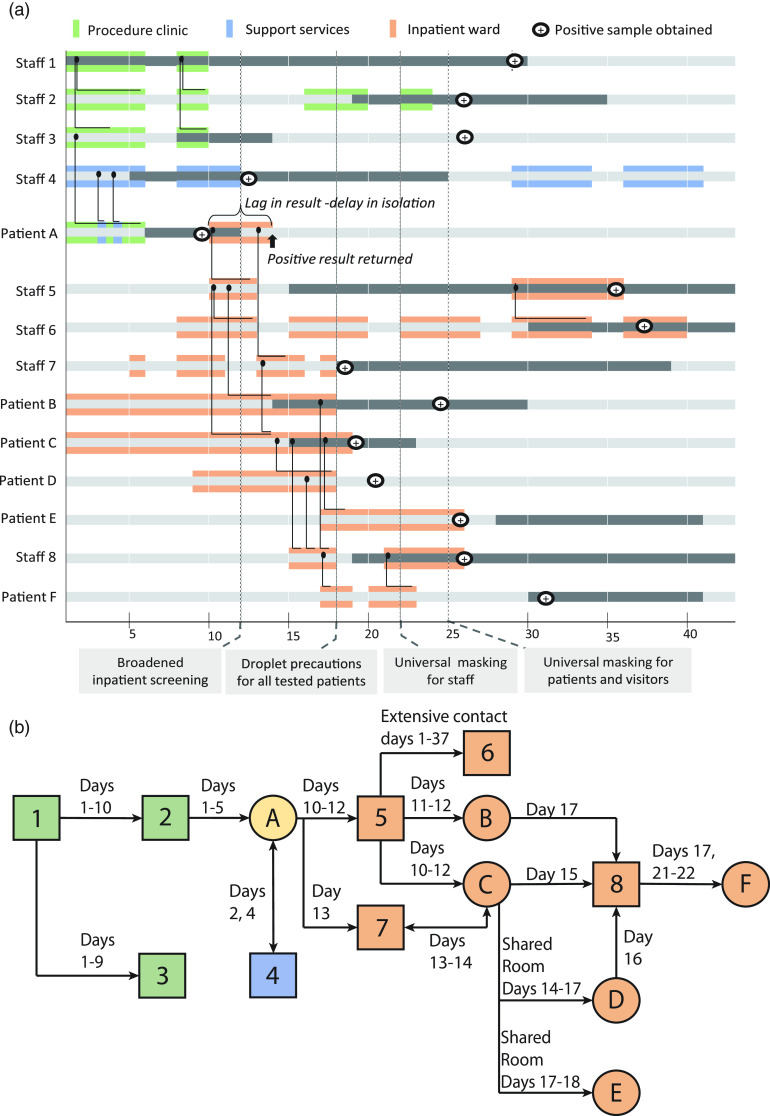
(A) Timeline shows transmission, exposures, symptoms, testing, and infection prevention measures in facility 1. Symptomatic periods for each individual are indicated by dark gray. Positive testing indicated on the date the sample was obtained. Colored shading indicates presence in the specified geographic location. Known interactions are indicated by solid black lines, with duration expressed as horizontal length of line. (B) Tree shows confirmed cases among patients (circles) and staff (squares) with arrows indicating likely direction of transmission. Days of interaction are indicated.

Across facilities 1 and 2 from days 1–40, 14 confirmed cases of healthcare-associated COVID-19 occurred among 250 exposed individuals (191 patients, 41 staff). Of these, 9 were linked to the general inpatient ward and 3 were linked to the procedural clinic. The index patient was associated with both areas and with a separate staff member who provided support services (Fig. 2). Moreover, 30 probable cases were identified (20 patients, 10 staff); of these, 18 tested negative for SARS-CoV-2 and 12 had no testing. We identified no transmission links between probable cases and confirmed COVID-19 cases. An additional 27 patients with community-acquired COVID-19 were admitted during the study period, but we found no transmission links from these individuals in our review of the records.

Among exposed patients, median age was 69 years; almost all were men (93.7%) and had at least 1 chronic medical condition (94.8%); the most common of these were hypertension (74.9%), diabetes mellitus (43.5%), and malignancy (41.9%) (Table 2).

**Table 2.** Demographic and Clinical Characteristics of Patients Evaluated During Outbreak Investigation

Characteristic	Patients Meeting Case Definition (N = 26)	Patients Not Meeting Case Definition (N=165)	All Patients Evaluated (N=191)
Age, median y (range)	73 (42–97)	69 (25–95)	69 (25–97)
Sex, male %	92.3	93.9	93.7
**Race, no. (%)**			
White	15 (57.7)	87 (52.7)	102 (53.4)
Black or African American	9 (34.6)	73 (44.2)	82 (42.9)
Other^a^	<5 (n/a)	5 (3.0)	7 (2.1)
**Underlying medical conditions, no. (%)**			
Hypertension	19 (73.1)	124 (75.2)	143 (74.9)
Cardiovascular disease	10 (38.5)	68 (41.2)	78 (40.8)
Diabetes mellitus	12 (46.2)	71 (43.0)	83 (43.5)
Chronic pulmonary disease	7 (26.9)	50 (30.3)	57 (29.8)
Chronic kidney disease	7 (26.9)	35 (21.2)	42 (22.0)
Malignancy	12 (46.2)	68 (41.2)	80 (41.9)
Smoking history	19 (73.1)	118 (71.5)	137 (71.7)
Obesity	8 (30.8)	60 (36.4)	68 (35.6)
Mean days of follow-up (range)	18 (2–41)	17.5 (0–41)	18 (0–41)

a Includes Hawaiian native or Pacific Islander, Asian, and Unknown by patient.

Among confirmed and probable cases of both patients and staff, 90.7% experienced fever, cough or shortness of breath (Table 3). The mean incubation period was 5.6 days (range, 1–14), and the mean length of follow-up from last potential exposure was 18 days (range, 0–41).

**Table 3.** Clinical Features of Patients and Staff Evaluated During the Outbreak Investigation

Symptoms and Signs	Confirmed and Probable Cases (N = 44), no. (%)
Fever^a^	25 (56.8)
Cough	14 (31.8)
Shortness of breath or hypoxia	16 (36.6)
Chills	<5 (n/a)
Fatigue	11 (25.0)
Myalgias	9 (20.5)
Headache	7 (15.9)
Anosmia	4 (9.1)
Sore Throat	11 (25.0)
Gastrointestinal symptoms^b^	11 (25.0)
Bilateral pulmonary infiltrates	6 (13.6)

a Fever included subjective fever and measured temperature >100.4°F (38°C).

b Gastrointestinal symptoms included abdominal pain, nausea, vomiting, or diarrhea.

All transmissions occurred among patients and staff with direct interactions or close proximity without use of medical or cloth masks, N95 respirators, or other personal protective equipment (PPE). Transmission between patients C, D, E and staff 8 occurred within a single inpatient room with an approximate area of 16 m^2^ (174 feet^2^) and a maximum distance of 2.8 m (9.1 feet) between beds. Although able to house 3 patients, the room had only 1 or 2 patients assigned throughout this period. None of the positive patients underwent aerosol-generating procedures (eg, continuous positive airway pressure therapy, nebulizer treatment, etc). The earliest date of potential transmission was day 1. The latest potential dates of staff-to-patient and staff-to-staff transmission were days 22 and 25, respectively.

### Surveillance

System-wide surveillance identified 40 SARS-CoV-2–positive patients admitted to our facilities during days 1–65 (Fig. 1). In addition, 6 healthcare-associated cases were identified during this period, of whom 4 met our definition of exposure. Also, 2 additional cases from the outbreak were tested at outside facilities and were identified outside the system-wide surveillance. The remaining 2 healthcare-associated cases occurred after implementation of universal masking and were unrelated to the outbreak. These cases were admitted on days 29 and 34, and neither had epidemiologic links to the outbreak locations or cases. One of these patients developed symptoms 5 days after admission, and 1 was asymptomatic and was tested 5 days after admission for discharge purposes. All potentially exposed staff were masked, and none developed symptoms or confirmed disease. Furthermore, 3 exposed patients subsequently tested negative for SARS-CoV-2.

Point-prevalence survey results are summarized in Table 1. All staff and patients meeting criteria for testing were included. No patients or staff refused testing or were lost to follow-up. No positive patient cases were identified, including in survey 3 (days 50–51) that tested inpatients on the previous outbreak ward in facility 1. Survey 2 (days 38–41) identified 5 employees positive for SARS-CoV-2 by rRT-PCR. All employees worked in separate areas and had no history of contact with each other. None of the employees had an epidemiologic link to a known COVID-19 patient at our facilities, and no SARS-CoV-2–positive patient was identified in their work areas. All employees who cared for the 5 positive cases tested negative.

## Discussion

Over 4 weeks, an outbreak of COVID-19 affected 14 staff members and patients. The outbreak began when a healthcare worker with recent out-of-state travel worked while symptomatic, serving as a source of infection for both patients and other healthcare workers. The outbreak spread when an infected patient, patient A, was transported from one facility to another and was admitted to an inpatient ward without droplet or contact precautions due to a delay in test results and reliance on an alternative explanation for symptoms in the setting of low COVID-19 community prevalence. There, he served as an ongoing source of infection.

No mode of transmission can be ruled out based on our data, but the pattern of infection in this outbreak suggests larger respiratory droplets or fomites as primary routes. Similar to a recent COVID-19 outbreak investigation at the University of California–Davis Medical Center, all infected individuals in the outbreak described here had prolonged direct encounters with another positive case without proper PPE.^20^ There was no spread between individuals in different rooms sharing an air handling system or in the direction of air flows out of rooms with infected patients (eg, to hallways). Of the 2 healthcare-associated cases that were not part of the outbreak, neither was ever on the same floor as the outbreak ward, in the procedural clinic, nor in an area sharing air handling systems with rooms housing COVID-19–positive patients.

All roommate pairs were positioned >3 m (9 feet) apart and were immobile, with limited if any use of the shared bathroom. Although 2 m (6 feet) is a frequent cutoff for physical distancing, the CDC *2007 Guideline for Isolation Precautions* notes the upper limit of droplet transmission is unresolved and may occur as far as ∼3.3 m (10 feet) from the source.^21^ Contamination of shared equipment or insufficient hand and environmental hygiene are other possible contributors to transmission, but our data raise concern that 2 m may be insufficient to prevent transmission between unmasked individuals with prolonged exposure in an enclosed space, consistent with known transmission characteristics of SARS-CoV and newer data on SARS-CoV-2.^22–24^

Early infection prevention interventions were insufficient to prevent or halt this outbreak. Daily inpatient screening was limited by variable application and inconsistent patient responses. Employee temperature checks began on day 4, but not all infected persons experience fever, and even those who do may have afebrile intervals. The results from survey 2 clearly demonstrate that infected employees were missed. In-house SARS-CoV-2 testing became available on day 14 but was initially limited to <5 tests per day. This testing had minimal impact on healthcare-associated transmission despite a 1-hour turnaround, allowing rapid isolation of infected patients.

The intervention best correlating with the end of the outbreak was implementation of universal masking on day 22, the last potential date of transmission from staff to patient. From this date forward, all staff wore medical face masks during patient contact and a cloth or medical face mask within the facility. One medical face mask was typically worn per shift on inpatient wards. Respirators were worn only on COVID-19 wards, and access outside those units was extremely limited. A broadened inpatient screening algorithm and droplet and contact precaution policy for tested patients may have also contributed to outbreak control, but multiple patients involved in the outbreak were not symptomatic or otherwise tested until after their transfer off the ward, and in-unit transmission continued after these policies were in place until the implementation of universal masking.

During the study period, no SARS-CoV-2 transmission events were documented involving patients or masked staff after implementation of universal masking, despite increased testing capacity and stable community prevalence. Although 2 cases met our definition of healthcare-associated COVID-19 after universal masking implementation, neither was epidemiologically linked to another positive patient or staff. One patient developed symptoms 5 days after admission, the median estimated incubation period for COVID-19, suggesting possible infection prior to admission.^25^ The other had no symptoms and may have been infected prior to admission. Neither case resulted in a transmission event.

Multiple point-prevalence surveys found no cases among inpatients in either of our facilities, even though 5 staff members from facility 2 tested positive. Thus, masking may have prevented transmission from healthcare workers to patients in our facility. The lack of contact between positive staff also indicate that masking may have been effective in preventing employee-to-employee transmission.

Our study has several limitations. This observational study includes multifactorial interventions and therefore cannot definitively prove the effectiveness of masking alone. We were unable to confirm transmission sources via viral genetic analysis; thus, some transmission may have occurred through alternative routes than those proposed. Due to resource limitations, we did not audit compliance with the universal masking policy. Finally, point-prevalence surveys were separated by 1–2 weeks and could have missed asymptomatic transmission events. However, we detected no symptomatic transmission events during these periods.

Healthcare institutions, and the community at large, face challenges in preventing SARS-CoV-2 transmission. Our data support universal masking as a means of preventing SARS-CoV-2 transmission in multiple healthcare settings. In the face of fluctuating infection rates, an uncertain supply of PPE, and ongoing questions regarding SARS-CoV-2 transmission, universal masking should be considered an important tool for COVID-19 infection prevention and control.
